# Microstructures and growth mechanisms of GaN films epitaxially grown on AlN/Si hetero-structures by pulsed laser deposition at different temperatures

**DOI:** 10.1038/srep16453

**Published:** 2015-11-13

**Authors:** Wenliang Wang, Weijia Yang, Yunhao Lin, Shizhong Zhou, Guoqiang Li

**Affiliations:** 1 State Key Laboratory of Luminescent Materials and Devices, South China University of Technology, Guangzhou 510640, China; 2 Engineering Research Center on Solid-State Lighting and its Informationisation of Guangdong Province, Guangzhou 510640, China

## Abstract

2 inch-diameter GaN films with homogeneous thickness distribution have been grown on AlN/Si(111) hetero-structures by pulsed laser deposition (PLD) with laser rastering technique. The surface morphology, crystalline quality, and interfacial property of as-grown GaN films are characterized in detail. By optimizing the laser rastering program, the ~300 nm-thick GaN films grown at 750 °C show a root-mean-square (RMS) thickness inhomogeneity of 3.0%, very smooth surface with a RMS surface roughness of 3.0 nm, full-width at half-maximums (FWHMs) for GaN(0002) and GaN(10
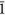
2) X-ray rocking curves of 0.7° and 0.8°, respectively, and sharp and abrupt AlN/GaN hetero-interfaces. With the increase in the growth temperature from 550 to 850 °C, the surface morphology, crystalline quality, and interfacial property of as-grown ~300 nm-thick GaN films are gradually improved at first and then decreased. Based on the characterizations, the corresponding growth mechanisms of GaN films grown on AlN/Si hetero-structures by PLD with various growth temperatures are hence proposed. This work would be beneficial to understanding the further insight of the GaN films grown on Si(111) substrates by PLD for the application of GaN-based devices.

Recently, III-nitride semiconductor materials such as GaN, AlN, InN, *etc.*, have attracted considerable attentions in the application of light-emitting diodes (LEDs), laser diodes (LDs), *etc*., due to their excellent physical and chemical properties, such as direct band, high thermal stability, *etc*^1^,^2^,^3^.

So far, GaN-based prepared based on sapphire substrates have already been commercialized thanks to the physical and chemical stabilities of sapphire at high temperature^4^,^5^. However, there are several shortcomings in the preparation of GaN-based devices on sapphire substrates. On the one hand, the thermal conductivity for sapphire is as small as 25 W/(m·K), which means that the generated heat in the devices can’t be conducted out timely and eventually reduce the performance and lifetime of GaN-based device^6^,^7^. On the other hand, the sapphire substrate is still of high cost. Therefore, the cost for the fabrication of GaN-based devices on sapphire substrates would be very expansive. In this regard, these two aspects make sapphire hard to meet the recent requirement of substrate for the growth of high-power and low-cost GaN-based devices.

Si, one of the most abundant elements on the earth, has excellent semiconducting properties and outstanding thermal conductivity, and therefore Si substrates can overcome the meeting problems of sapphire substrates in the preparation of GaN-based devices^8^,^9^. First, the thermal conductivity of Si is as high as 130 W/(m·K), which is 5 times of that for sapphire substrates^6^,^10^. Therefore, the generated heat in the GaN-based devices can be conducted out timely, and eventually benefits to the performance of GaN-based devices. Second, the Si substrates are available of large size and low cost, which would significantly reduce the cost of fabrication GaN-based devices on Si substrates^8^. However, there are still several issues to be addressed for the fabrication of GaN-based devices on Si substrates. On the one hand, the lattice and coefficient of thermal expansion (CTE) mismatches between Si and GaN are as high as 16.9% and −115%, respectively, which means that high dislocation density will form during the film epitaxial growth and eventually lead to the failure of epitaxial growth^11^,^12^. On the other hand, it is hard to directly grow GaN films on Si due to the serious reactions between Si and Ga^9^.

To date, various materials such as AlN, Sc_2_O_3_, SiC, *ect*., have been used as buffer layer to hamper the chemical reactions between Si and Ga and to reduce the lattice and CTE mismatches between Si and GaN for the growth of GaN films on Si substrates^9^,^13^,^14^. By now, the GaN films on Si substrates are usually grown by metallic-organic chemical vapor deposition (MOCVD), which usually need high growth temperature and thereby high energy consumption^15^,^16^. Furthermore, there is an interfacial layer of SiN_x_ existing between buffer layers and Si substrates during the epitaxial growth of III-nitride films by MOCVD due to the interfacial reactions between N from ammonia or nitrogen plasma and Si from the substrate^17^. Actually, many dislocations are formed in this interfacial layer, which is detrimental to the subsequent growth. To overcome this issue, pulsed laser deposition (PLD) is deployed. PLD can supply high-energy and assist the migration of the precursors on the surface, and therefore make the films growth at low temperature possible^18^,^19^. The low temperature growth not only can effectively suppress the interfacial reactions between films and substrates, but also reduces the energy consumption^20^,^21^.

So far, GaN films grown on Si substrates by traditional PLD have been achieved. For example, J. Ohta *et al.* reported the growth of GaN films on Si substrates with AlN buffer layer by traditional PLD and used *in-situ* reflection high energy electron diffraction (RHEED) and X-ray photoelectron spectroscopy (XPS) to study the properties of as-grown GaN films^22^. X. L. Tong *et al.* also deployed the X-ray diffraction (XRD) and photoluminescence (PL) to characterize the structural and optoelectronic properties of GaN films on Si(111)substrates grown by traditional PLD^23^. However, the as-grown GaN films grown by traditional PLD usually show poor thickness homogeneity due to the highly directional distribution of the precursors on the substrates^24^,^25^,^26^,^27^. Meanwhile, the surface morphology, crystalline quality, and interfacial properties of AlN/GaN hetero-interfaces grown by PLD, as well as the growth mechanism for GaN films grown on AlN/Si hetero-interfaces, lack thorough study.

In this work, we report on the epitaxial growth of GaN films with homogeneous thickness distribution on the homogeneous AlN/Si hetero-structures that were achieved in our previous work by PLD with optimized laser rastering program^28^,^29^. On the one hand, the programmed and optimized movement of the rastering mirror intentionally changes the incident angles of precursors toward the substrates produced by the each laser pulse, resulting in a statistically homogeneous distribution of precursors on the substrate and thereby the homogeneous thickness of as-grown GaN films can be achieved^26^,^27^,^28^,^29^. On the other hand, the surface morphology, crystalline quality, and interfacial property of as-grown ~300 nm-thick GaN films with the growth temperature ranging from 550 to 850 °C are studied systematically. Furthermore, based on the characterizations, the corresponding growth mechanisms of GaN films grown on AlN/Si hetero-structures with various growth temperatures are hence proposed. This work would be beneficial to understanding the further insides of the growth of GaN films on Si substrates by PLD for the future application of GaN-based devices.

The as-received 2-inch Si(111) substrates were firstly cleaned by H_2_SO_4_ : H_2_O_2_ : H_2_O (3:1:1) and buffered-oxide-etch (BOE) HF (20:1) to obtain an oxide-free and hydrogen terminated Si surfaces. Subsequently, the as-cleaned Si(111) substrates were transferred into an ultra-high vacuum (UHV) growth chamber with a background pressure of 3.0 × 10^−10^  Torr before the Si(111) substrates were taken a degassing treatment in an UHV load-lock with a background pressure of 1.0 × 10^−8^ Torr. Before the epitaxial growth, the as-transferred Si(111) substrates were taken a 60 min annealing process at 850 °C to remove the residual contaminations on the Si(111) surfaces. Afterwards, the homogeneous ~30 nm-thick AlN films were found to be an optimized growth condition for GaN growth and the AlN films were grown with optimized laser rastering program reported by H. Yang, *et al.* to achieve AlN/Si hetero-structures^20^,^28^. After epitaxial growth of AlN/Si hetero-structures, the distance between the target and substrate was changed from 8 cm to 5 cm. During the GaN epitaxial growth, high-purity nitrogen with the optimized pressure of 10 mTorr was supplied through the inert gas purifier and the radio-frequency plasma radical generator was operated at 500 W. The laser energy density was set at 3.0 J/cm^2^ with the pulse repetition rate of 30 Hz to grow homogeneous ~300 nm-thick GaN films on AlN/Si hetero-structures at growth temperature ranging from 550 to 850 °C. Subsequently, the ~300 nm-thick GaN films are grown on these AlN/Si hetero-structures with various laser rastering programs^20^,^28^. Sample A, is grown without the utilization of the laser rastering program, Sample B is grown with identical laser rastering rates of 100 steps/s for each segment along the Ga target radius. While for Sample C, the optimized laser rastering program is utilized. In this case, the Ga target radius is divided into 4 segments equally compared with that for the growth of AlN with 3 segments to obtain homogenous thickness GaN films, as shown in Fig. 1a,b, respectively^20^. As the thickness of as-grown GaN films is ~300 nm-thick, which is much thicker than AlN films with ~30 nm-thick, the laser rastering parameters need to be further optimized to obtain GaN films with homogeneous thickness distribution. Therefore, as for GaN growth, the optimized laser rate is 100 steps/s for S1, and is gradually reduced to 75, 50, and 25 steps/s for S2, S3, and S4, respectively.

The as-grown ~300 nm-thick GaN films were characterized by white-light interferometry (Y-Wafer GS4-GaN-R-405), *in-situ* RHEED, polarized light microscopy (PLM, OLYMPUS, BX51M), scanning electron microscopy (SEM, Nova Nano SEM 430 Holland), atomic force microscopy (AFM, Bruker Dimension Edge, American), high-resolution XRD (Bruker D8 X-ray diffractometer with Cu Kα1 X-ray source *λ* = 1.5406 Å) and high-resolution transmission electron microscopy (HRTEM, JEOL 3000F). As for the TEM characterization, samples of GaN films grown on AlN/Si(111) hetero-structures were made by mechanical polishing to ~50 μm, followed by low-energy and low-angle ion milling (Fischione 1010 Low Angle Ion Milling & Polishing System). At first, the low-energy and low-angle for ion milling were set as 5 keV and 10°, respectively. When the hole appeared, the low-energy and low-angle for ion milling were reduced to be 4 keV and 4-5°, respectively, ending up with the sample edge thickness of about 20 nm. The cross-section samples were then put into a JEOL 3000F field emission gun TEM working at a voltage of 300 kV, which gives a point to point resolution of 0.17 nm. The electron energy loss spectroscopy (EELS) attached to the TEM was deployed to evaluate the GaN/AlN hetero-structures.

It is known that the white-light interferometry requires that the thickness of as-grown films should usually be >200 nm^28^,^29^. Therefore, the thickness of as-grown AlN films obtained in this work is calculated by grazing incident X-ray reflectivity measurement reported in our previous work^28^. The thickness of as-grown GaN films are calculated by white-light interferometry. It should be noted that the thickness mentioned in GaN film system has excluded the 30 nm-thick AlN films in the followings. Fig. 2 shows the photographs of ~300 nm-thick GaN films grown on AlN/Si hetero-structures and their corresponding thickness distribution over the 2 inch-diameter measured by white-light interferometry. Fig. 2a is photograph for GaN films grown with traditional PLD technology without utilization of laser rastering program, where very inhomogeneous GaN films can be found in Fig. 2b. In this case, the root-mean-square (RMS) thickness inhomogeneity is calculated to be as high as 12.1%. As the identical rastering program is used in Sample B, as shown in Fig. 2c, the RMS thickness inhomogeneity of as-grown ~300 nm-thick GaN films is reduced to be 8.6% calculated by white-light interferometry shown in Fig. 2d, which is still not good enough. When the optimized laser rastering program is deployed, the RMS thickness inhomogeneity of as-grown ~300 nm-thick GaN films is further decreased to be 3.0%, as illustrated in Fig. 2e,f. In this regard, the application of an optimized laser rastering program integrated in PLD can greatly improve the homogeneity of as-grown GaN films on AlN/Si hetero-structures with homogeneous thickness distribution. This may be ascribed to the utilization of optimal laser program, where the movement of the rastering mirror intentionally changes the locations on and the incident angles towards the substrates, and eventually results in the homogeneous distribution of precursors on the substrates^20^,^26^,^27^.

During the epitaxial growth, the *in-situ* RHEED is adopted to monitor the whole growth process. Fig. 3a is the RHEED patterns for ~30 nm-thick AlN films grown on Si substrates at 750 °C, where sharp and streaky patterns can be found clearly, indicating that AlN films with very smooth surface have been obtained and is good for the subsequent growth of GaN films. After growth of ~30 nm-thick AlN films, ~300 nm-thick GaN films are then grown on AlN/Si substrates. Fig. 3b shows the ~300 nm-thick GaN films grown with growth temperature of 500 °C, where nothing in this pattern can be seen. This demonstrates the very rough surface for ~300 nm-thick GaN films grown at 500 °C. As the growth temperature is increased to 750 °C, very clear and linear RHEED patterns for ~300 nm-thick GaN films grown at 750 °C can be observed clearly, as shown in Fig. 3c, which reveals the very smooth GaN surfaces. These RHEED patterns are striking contrast to the spotty patterns for GaN films grown on Si substrates with AlN buffer layer reported before^22^. These results confirm the smoother surface of GaN films grown in this work. However, when the growth temperature is further raised to 850 °C, the spotty RHEED patterns for these as-grown ~300 nm-thick GaN films are obtained, as shown in Fig. 3d, indicating the decrease of GaN surface. On the one hand, the RHEED patterns of AlN and GaN obtained in this work agree with those of AlN and GaN along [11
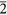
0] direction^22^. On the other hand, the diffraction patterns of AlN and GaN along [11
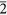
0] direction are obtained under the same conditions. These two aspects make us to conclude that an in-plane epitaxial relationship of AlN[11
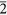
0]//GaN[11
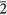
0] between AlN and GaN can be determined^18^,^19^. By the combination of our previous results^18^,^19^,^28^, an in-plane epitaxial relationship of Si[1
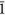
0]//AlN[11
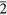
0]//GaN[11
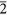
0] can be obtained for GaN films grown on Si substrates with AlN buffer layer.

The SEM and AFM are adopted to identify the surface morphology of as-grown ~30 nm-thick AlN films in AlN/Si hetero-structures and as-grown ~300 nm-thick GaN films, respectively. Fig. 4a shows the SEM and AFM images for ~30 nm-thick AlN films, where one can find very smooth AlN surface with a RMS surface roughness of 2.3 nm. Meanwhile, the height profiles along a straight dashed line on the surface are provided in Fig. 4b, which also confirms the very smooth AlN surface and is consistent with the SEM result. Fig. 4c reveals typical SEM and AFM image for ~300 nm-thick GaN films grown at 550 °C, where very rough GaN surface with a RMS surface roughness of 102.1 nm is obtained. Furthermore, the large islands distributed on the GaN surface are in the diameter of about 500 nm to 1 μm, which is further confirmed by the height profiles, as shown in Fig. 4d. As the GaN growth temperature is increased to be 750 °C, very smooth GaN surface with a RMS surface roughness of 3.0 nm is obtained, as indicated in Fig. 4e. This surface roughness is different from that of GaN films grown on Si substrates by PLD with a RMS surface roughness of 3.3 nm^23^. We ascribed to the optimized GaN growth conditions, especially, the growth temperature. Actually, the grain size of as-grown films is measured to be 100-200 nm by the height profiles shown in Fig. 4f. However, when the growth temperature is further increased to 850 °C, the surface morphology of as-grown ~300 nm-thick GaN films becomes slightly poorer. Fig. 4g shows that there are many small islands distributed on GaN surface, and the RMS surface roughness of as-grown GaN films is calculated to be 6.5 nm by AFM. The height profiles shown in Fig. 4h confirm that the size of islands is in the range of 100-500 nm. To conclude, the results may be ascribed to the suitable temperature growth of GaN films. When the growth temperature is too low, the GaN precursors can’t receive enough energy for the migration to their lowest energy crystal positions, which are superimposed by additional GaN precursors and eventually leads to the formation of large islands^30^. When the growth temperature is too high, the active AlN may react with high-energy Ga plasmas and lead to formation of AlGaN layer during the initial growth^31^,^32^,^33^,^34^. In this layer, there are many defects are formed, which is detrimental to the subsequent growth of GaN films, and eventually rough the GaN surfaces.

XRD is deployed to study the structural property of as-grown ~300 nm-thick GaN films. Fig. 5a is a typical XRD 2*θ*-*ω* scan for GaN films grown on AlN/Si hetero-structures at various temperatures. The peaks located at 2*θ* = 34.56° and 72.64° are the diffractions of GaN(0002) and GaN(0004), respectively^19^; and the peaks observed at 2*θ* = 36.02° and 76.50°are the diffractions of AlN(0002) and AlN(0004), respectively^18^. While the peaks found at 2*θ* = 28.44°, 58.90° and 95.02°are the diffractions of Si(111), Si(222) and Si(333), respectively^28^. In this regard, the out-of-plane epitaxial relationship of GaN(0001)//AlN(0001)//Si(111) is therefore can be determined. Furthermore, one can clearly find that the intensity of GaN(0002) and GaN(0004) for ~300 nm-thick GaN films is gradually raised as the GaN growth temperature increases from 550 to 750 °C. However, as the GaN growth temperature is further increased to 850 °C, the intensity of GaN(0002) and GaN(0004) for ~300 nm-thick GaN films is slightly reduced. Based on these results, one can speculate that the highest crystalline quality of ~300 nm-thick GaN films is grown at 750 °C. Meanwhile, the peak of GaN(0002) grown at 750 °C is sharper than that reported before^22^,^23^, revealing the higher crystalline quality of GaN obtained in this work. To further evaluate the in-plane epitaxial relationship between GaN films and Si substrates, XRD *φ* scan both for the films and substrates is conducted. Fig. 5b reveals the typical *φ* scans of Si(1
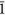
3), AlN(11
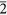
2), and GaN(11
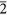
2), where six-fold rotational peaks with an internal of 60° can been clearly identified. On the one hand, these results confirm the single-crystalline hexagonal GaN films have been grown on Si(111) substrates^27^,^33^,^34^. On the other hand, an in-plane epitaxial relationship is determined to be GaN[11
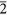
0]//AlN[11
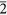
0]//Si[1
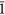
0]^19^,^31^,^32^, which is well consistent with the result of RHEED measurement. X-ray rocking curve (XRC) is a normal method to determine the crystalline quality of GaN films, because it is demonstrated that the full-width at half-maximum (FWHM) of GaN(0002) and GaN(10
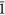
2) XRC is related to the dislocation density in as-grown GaN films^2^,^32^. Fig. 5c,d show the typical GaN(0002) and GaN(10
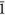
2) XRCs for ~300 nm-thick GaN films grown on Si substrates at 750 °C. From Fig. 5c,d, the FWHM for GaN(0002) and GaN(10
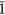
2) XRCs is measured to be 0.7° and 0.8°, respectively. Furthermore, the temperature dependence of FWHM for GaN(0002) and GaN(10
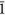
2) XRC is also studied, as shown in Fig. 5e. One can clearly identify that the FWHM for GaN(0002) and GaN(10
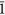
2) XRC is as large as 3.2° and 4.1° at growth temperature of 550°C, respectively; and is monotonously decreased to 0.7° and 0.8° at growth temperature of 750 °C, respectively. These results reveal the increase in crystalline quality of GaN films as the growth temperature raises from 550 to 750 °C. However, when the growth temperature is further increased to be 850 °C, the crystalline quality of as-grown ~300 nm-thick GaN films become poorer with the FWHM for GaN(0002) and GaN(10
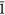
2) XRC of 1.5° and 1.7°, respectively. These results can be explained to be the suitable growth temperature by PLD. When the GaN is grown at too low temperature, the GaN can’t not receive enough energy for the nucleation, where many dislocation are found during the initial growth^30^. As for the GaN grown too high temperature, the Ga plasmas produced by pulsed laser may react with AlN and results in the formation of many dislocations during the initial growth^31^,^32^,^33^,^34^. Actually, the dislocations in these two cases would extend into the subsequent growth films, and eventually lead to poor-quality of as-grown GaN films.

Reciprocal space mapping (RSM) is utilized to study the strain state in as-grown films. Fig. 6a shows the GaN(10
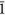
5) RSM of ~300 nm-thick GaN films grown at 750 °C, from which the lattice parameters for GaN films are measured to be *a* = 0.32313 nm and *c* = 0.51792 nm^2^,^35^. These results reveal that as-grown ~300 nm-thick GaN films at 750 °C are about 0.13% tensile along their *a* axis and about 1.31% compressive along their *c* axis, because the lattice parameters for fully relaxed GaN are *a* = 0.31895 nm and *c* = 0.51860 nm^2^,^35^,^36^,^37^. The strain state for GaN films grown with various temperatures is also studied, as shown in Fig. 6b. As for the ~300 nm-thick GaN films grown on 550 °C, the GaN(10
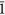
5) peak can’t be detected in this work, and therefore the strain state for these GaN films can’t be got from GaN(10
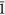
5) RSM. By using the same method, the ~300 nm-thick GaN films grow at 650 °C are about 0.58% tensile along their *a* axis and about 3.11% compressive along their *c* axis. While for the GaN films grow at 850 °C, the as-grown ~300 nm-thick GaN films are about 0.38% tensile along their *a* axis and about 2.51% compressive along their *c* axis. We attribute these results to the various growth temperatures. The suitable growth temperature is beneficial to nucleation of GaN films during the initial growth, which can rapidly release the stress in as-grown GaN films during the subsequent growth^36^.

Cross-sectional TEM is adopted to study the interfacial properties of as-grown GaN films. Fig. 7a is a typical cross-sectional TEM for GaN/AlN, where GaN films are grown at 550 °C. From Fig. 7a, one can clearly find that there is a ~3 nm-thick interfacial layer existing between AlN films and GaN films. The EELS is deployed to determine the chemical composition of this interfacial layer, as indicated in Fig. 7b. The sharply increasing of Ga and decreasing of Al mean that the layer is GaN layer. However, the GaN is interfacial layer and is slightly different from the uplayer GaN. The formation of this interfacial layer may be ascribed to the fact that GaN precursors are hard to be nucleated on AlN during the initial growth due to the inadequate energy of GaN precursors grown at this stage, which leads to the formation of GaN interfacial layer to release the stress between AlN and GaN^27^. When the growth temperature of GaN films are increased to 750 °C, very sharp and abrupt GaN/AlN hetero-interfaces are formed shown in Fig. 7c. This may be due to the fact that GaN precursors have enough energy for the migration on AlN surface, and the interfacial reactions between AlN and GaN are effectively suppressed^30^,^31^,^32^. Fig. 7d is the selected area electron diffraction (SAED) patterns for GaN grown on AlN/Si hetero-structures, where we can find the sharp and abrupt patterns for all of them. However, when the GaN films are grown on AlN at high temperature of 850 °C, we find that there is a ~2 nm-thick interfacial layer existing between GaN and AlN illustrated in Fig. 7e, which is determined to be AlGaN by EELS measurement as shown in Fig. 7f. The formation of AlGaN may be the reactions between AlN and Ga plasmas during the high temperature growth^30^,^31^,^32^. Conclusively, GaN films grown at suitable growth temperature of 750 °C reveal the most excellent interfacial properties.

Based on the above mentioned, we can therefore conclude the growth mechanisms of GaN films grown on AlN/Si hetero-structures at various growth temperatures. As for GaN films grown at too low temperature of 550 °C, the GaN precursors can’t receive enough energy for the migration on the AlN surface and is detrimental to the GaN nucleation during the initial growth^38^,^39^. To release the stress between AlN and GaN due to the lattice and CTE mismatches, the ~3 nm-thick GaN interfacial layer is formed. Actually, many dislocations are formed in this interfacial layer, which may extend into the subsequent GaN films and eventually lead to poor-quality GaN films with many large islands distributed on the GaN surface and poor crystalline quality, as shown in Fig. 8a^38^,^39^. When the growth temperature is raised from 550 to 750 °C, the quality of GaN films is improved monotonously. As for GaN films grown at 750 °C, the interfacial reaction between GaN and AlN can be effectively suppressed, and GaN has enough energy for the nucleation on AlN layer^38^,^39^. Both of these two aspects enhance the crystalline quality and surface roughness of GaN films, as shown in Fig. 8b. However, when the growth temperature is further increased, the quality of GaN films become slightly poorer. As for GaN grown too high temperature, the interfacial reactions between Ga plasmas and AlN may lead to the formation of interfacial layer of AlGaN, where many dislocations are formed, and may propagate into the subsequent growth films and lead to poor-quality films with several small islands and poor crystalline quality, as shown in Fig. 8c^38^,^39^.

In summary, GaN films with homogeneous thickness distribution have been grown on AlN/Si hetero-structures by PLD with optimized laser rastering program. The effect of GaN growth temperature on the surface morphology, crystalline quality, and interfacial property of ~300 nm-thick GaN films is studied in detail. It is found that the RMS surface roughness, FWHMs for GaN(0002) and GaN(10
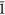
2) XRCs, and interfacial layer thickness for ~300 nm-thick GaN films, which are 102.1 nm, 3.2° and 4.1°, and 3 nm, respectively, at growth temperature of 550 °C; are gradually improved to 3.0 nm, 0.7° and 0.8°, and 0 nm, respectively, at growth temperature of 750 °C; and then are deteriorated to 6.5 nm, 1.5° and 1.7°, and 2 nm, respectively, at growth temperature of 850 °C. It is clear to find that as the the growth temperature increases from 550 to 850 °C, the surface morphology, crystalline quality, and interfacial property of as-grown ~300 nm-thick GaN films are gradually improved at first and then decreased. The growth mechanisms for GaN films grown on AlN/Si hetero-structures are hence proposed based on the characterizations. This work is of paramount importance for the fabrication of low-cost GaN-based devices on Si substrates. In this regard, future work should be focused on the preparation of GaN-based devices with these GaN films.

## Additional Information

**How to cite this article**: Wang, W. *et al.* Microstructures and growth mechanisms of GaN films epitaxially grown on AlN/Si hetero-structures by pulsed laser deposition at different temperatures. *Sci. Rep.* **5**, 16453; doi: 10.1038/srep16453 (2015).

## Figures and Tables

**Figure 1:**
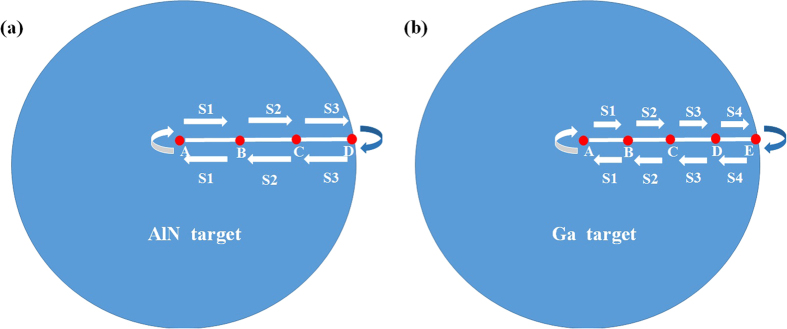
Schematic diagrams of the programmable movement of laser rastering mirror for the epitaxial growth of (**a**) AlN and (**b**) GaN films with laser rastering technique.

**Figure 2:**
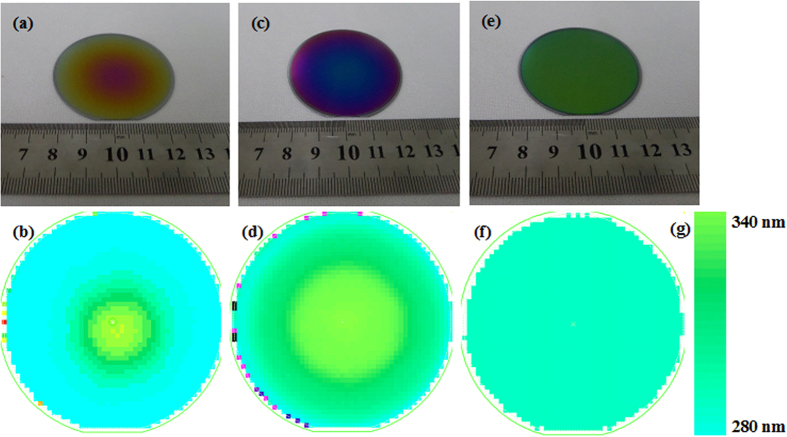
(**a**) Typical photograph of the GaN films grown on AlN/Si hetero-structures at 750 °C with traditional laser rastering program and (**b**) its corresponding distribution of thickness. (**c**) Typical photograph of the GaN films grown on AlN/Si hetero-structures at 750 °C with identical laser rastering program and (**d**) its corresponding distribution of thickness. (**e**) Typical photograph of the GaN films grown on AlN/Si hetero-structures at 750 °C with optimized laser rastering program and (**f**) its corresponding distribution of thickness. (**g**) The color scale of thickness distribution image.

**Figure 3:**
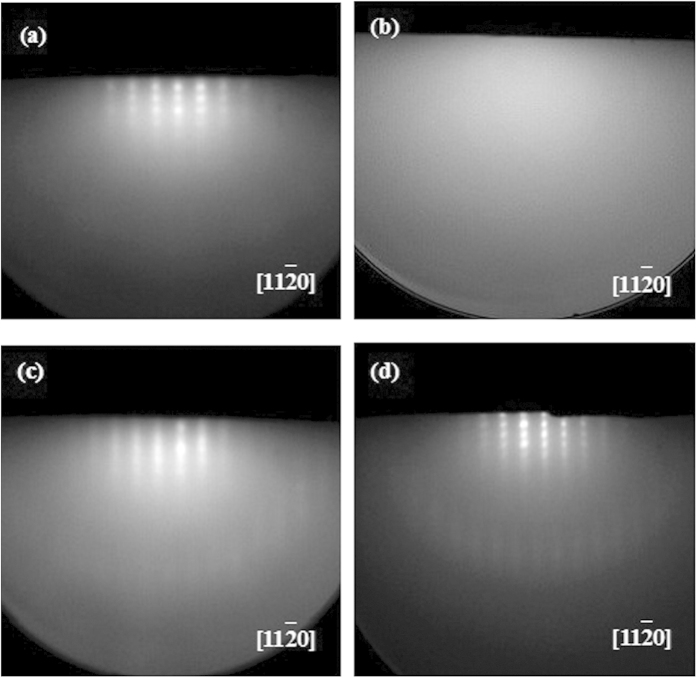
RHEED patterns for (**a**) ~30 nm-thick AlN films grown at 750 °C, and ~300 nm-thick GaN films grown on Si(111) substrates at (**b**) 550, (**c**) 750, and (**d**) 850 °C, respectively.

**Figure 4:**
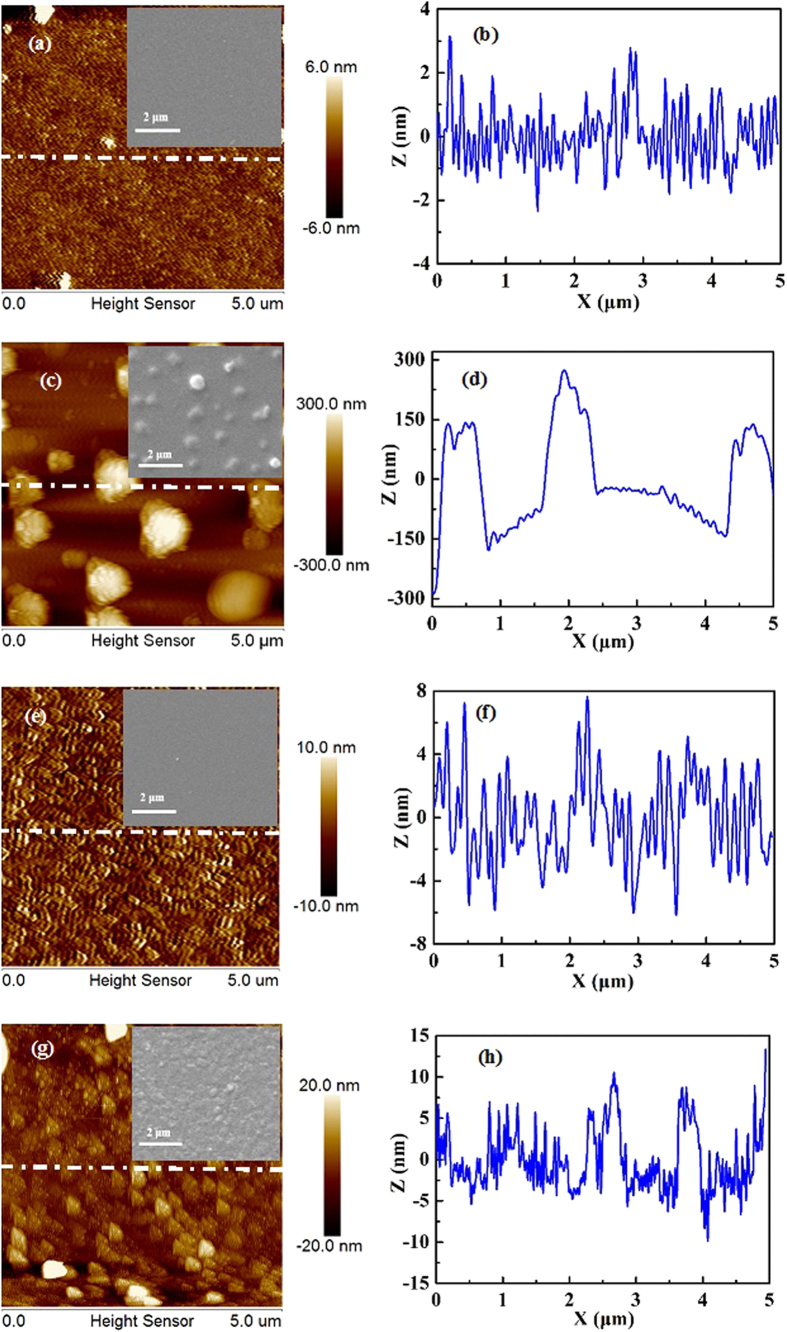
(**a**) SEM and AFM images for ~30 nm-thick AlN films grown on Si(111) substrates grown at 750 °C and (**b**) the height profiles along a straight dashed line on the surface. (**c**) SEM and AFM images for ~300 nm-thick GaN films grown on AlN/Si hetero-structures at 550 °C and (**d**) the height profiles along a straight dashed line on the surface. (**e**) SEM and AFM images for ~300 nm-thick GaN films grown on AlN/Si hetero-structures at 750 °C and (**f**) the height profiles along a straight dashed line on the surface. (**g**) SEM and AFM images for ~300 nm-thick GaN films grown on AlN/Si hetero-structures at 850 °C and (**h**) the height profiles along a straight dashed line on the surface.

**Figure 5:**
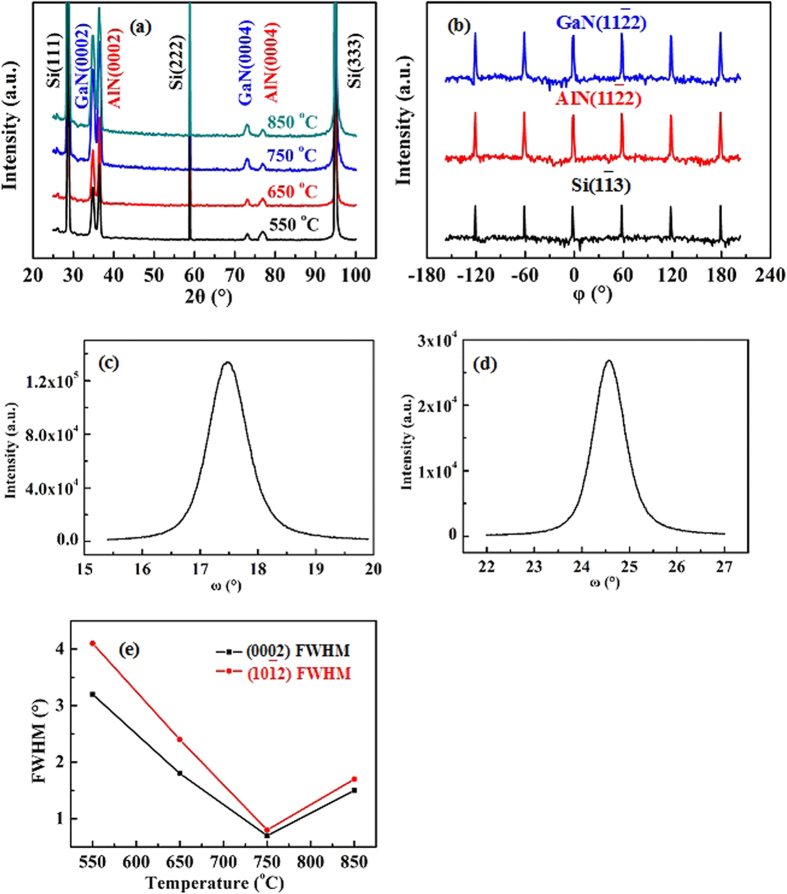
(**a**) Temperature dependence of typical XRD 2*θ* − *ω* scan for ~300 nm-thick GaN films grown on AlN/Si hetero-structures at various temperatures ranging from 550 to 850 °C. (**b**) XRD *φ* scans for Si(1
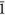
3), AlN(11
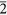
2) and GaN(11
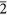
2), respectively. XRCs of (c) GaN(0002) and (**d**) GaN(10
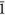
2), respectively. (**e**) Temperature dependence of FWHMs of GaN(0002) and GaN(10
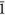
2) for ~300 nm-thick GaN films grown on AlN/Si hetero-structures at various temperatures ranging from 550 to 850 °C.

**Figure 6:**
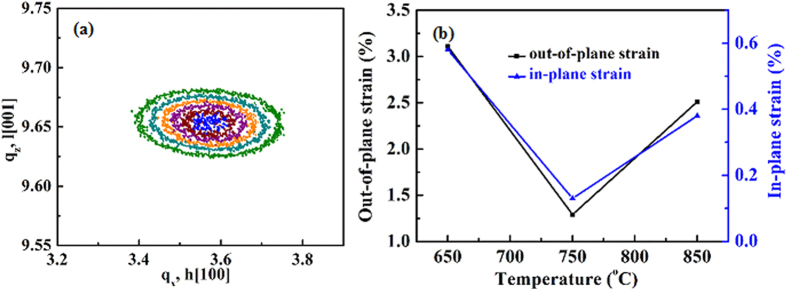
(**a**) RSMs of GaN(10
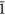
5) and (**b**) the strains of ~300 nm-thick GaN films grown on the AlN/Si hetero-structures at various temperatures ranging from 550 to 850 °C.

**Figure 7:**
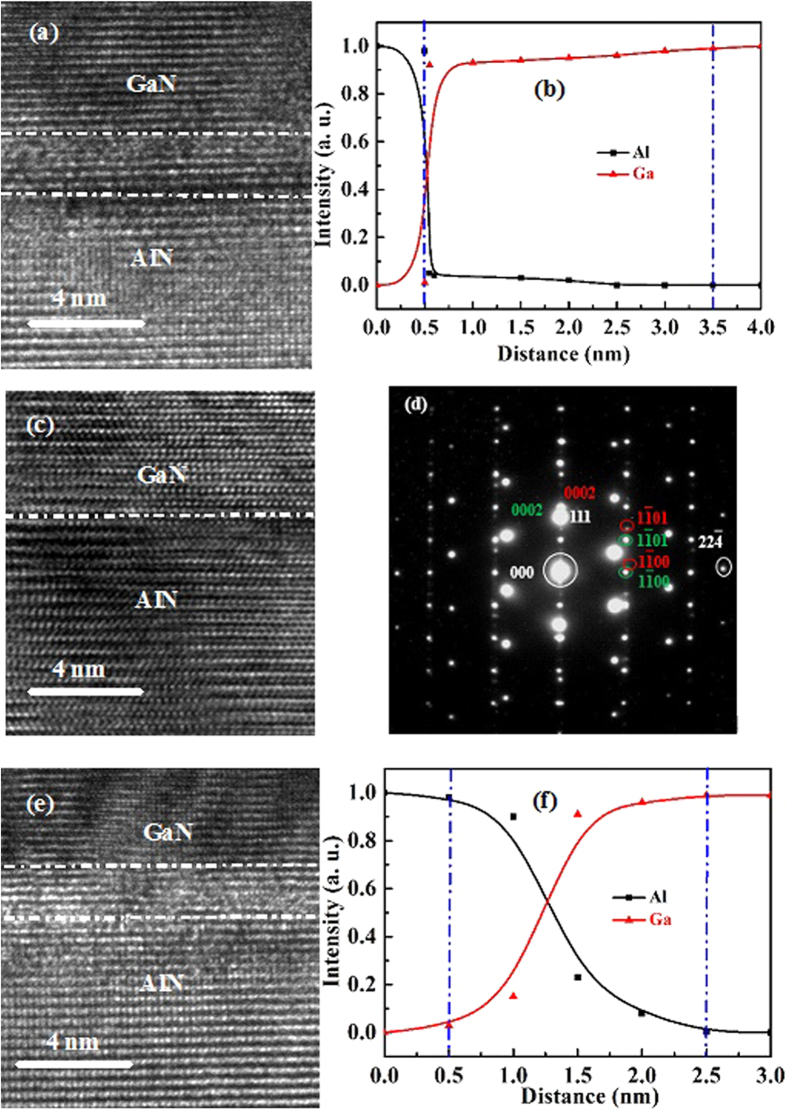
Cross-sectional high-resolution TEM images for GaN film grown on AlN/Si hetero-interface. (**a**) High-resolution TEM image for AlN/GaN hetero-interface grown at 550 °C and (**b**) its corresponding EELS curves for the hetero-interface. (**c**) High-resolution TEM image for AlN/GaN hetero-interface grown at 750 °C and (**d**) its corresponding SAED pattern where the spots marked in white, red, and green correspond to plane of Si, AlN, and GaN, respectively. (**e**) High-resolution TEM image for AlN/GaN hetero-interface grown at 850 °C and (**f**) its corresponding EELS curves for the hetero-interface.

**Figure 8:**
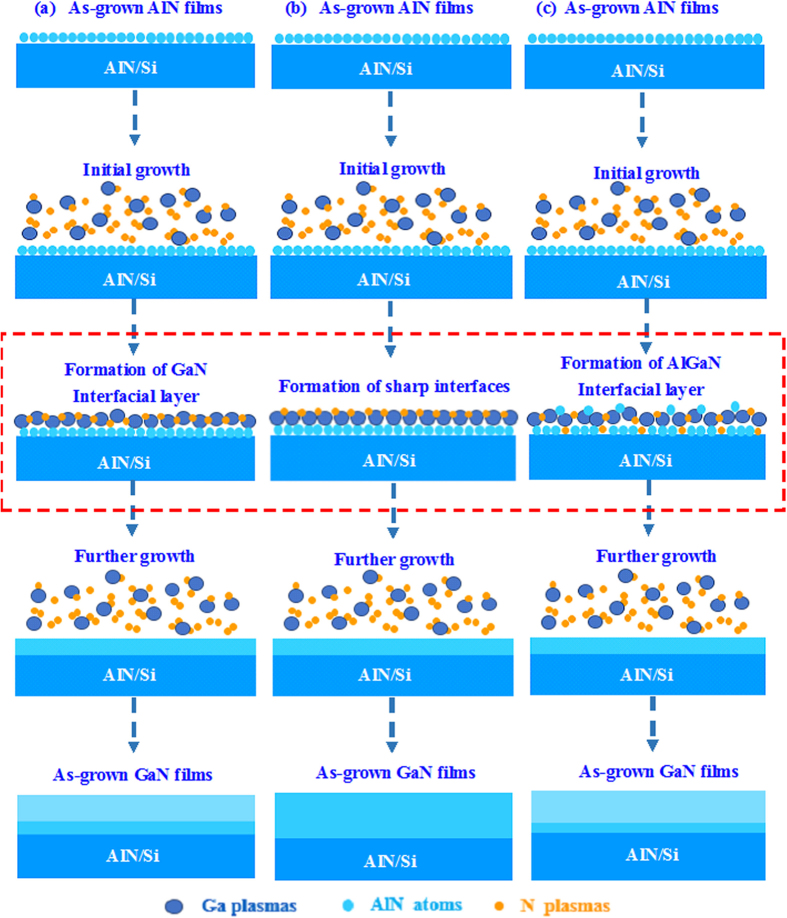
Schematic diagrams for the GaN films grown on AlN/Si hetero-structures at (**a**) 550, (**b**) 750, and (**c**) 850 °C, respectively.
